# Cryoballoon pulmonary vein isolation-mediated rise of sinus rate in patients with paroxysmal atrial fibrillation

**DOI:** 10.1007/s00392-020-01659-0

**Published:** 2020-05-13

**Authors:** Lara Wagner, Fabrice F. Darche, Dierk Thomas, Patrick Lugenbiel, Panagiotis Xynogalos, Svenja Seide, Eberhard P. Scholz, Hugo A. Katus, Patrick A. Schweizer

**Affiliations:** 1 Department of Cardiology, University Hospital Heidelberg, INF 410, 69120 Heidelberg, Germany; 2 Heidelberg Center for Heart Rhythm Disorders (HCR), University Hospital Heidelberg, Heidelberg, Germany; 3 DZHK (German Centre for Cardiovascular Research), Partner Site Heidelberg/Mannheim, University of Heidelberg, Heidelberg, Germany; 4 Institute of Medical Biometry and Informatics, University of Heidelberg, INF 130.3, 69120 Heidelberg, Germany

**Keywords:** Pulmonary vein isolation, Cryoballoon ablation, Atrial fibrillation, Sinus node, Heart rate, Autonomic nervous system

## Abstract

**Background:**

Modulation of the cardiac autonomic nervous system by pulmonary vein isolation (PVI) influences the sinoatrial nodal rate. Little is known about the causes, maintenance and prognostic value of this phenomenon. We set out to explore the effects of cryoballoon PVI (cryo-PVI) on sinus rate and its significance for clinical outcome.

**Methods and results:**

We evaluated 110 patients with paroxysmal atrial fibrillation (AF), who underwent PVI using a second-generation 28 mm cryoballoon by pre-, peri- and postprocedural heart rate acquisition and analysis of clinical outcome. Ninety-one patients could be included in postinterventional follow-up, indicating that cryo-PVI resulted in a significant rise of sinus rate by 16.5% (+ 9.8 ± 0.9 beats/min, *p* < 0.001) 1 day post procedure compared to preprocedural acquisition. This effect was more pronounced in patients with initial sinus bradycardia (< 60 beats/min.) compared to patients with faster heart rate. Increase of rate was primarily driven by ablation of the right superior pulmonary vein and for a subset of patients, in whom this could be assessed, persisted ≥ 1 year after the procedure. AF recurrence was neither predicted by the magnitude of the initial rate, nor by the extent of rate change, but postprocedural sinus bradycardia was associated with higher recurrence of AF in the year post PVI.

**Conclusions:**

Cryo-PVI causes a significant rise of sinus rate that is more pronounced in subjects with previous sinus bradycardia. Patient follow-up indicates persistence of this effect and suggests an increased risk of AF recurrence in patients with postprocedural bradycardia.

**Graphic abstract:**

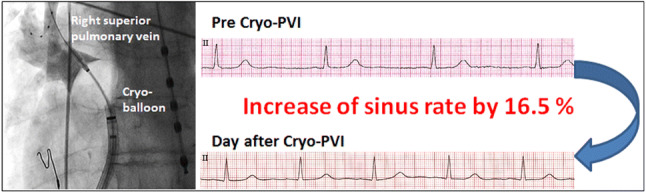

**Electronic supplementary material:**

The online version of this article (10.1007/s00392-020-01659-0) contains supplementary material, which is available to authorized users.

## Introduction

Patients with paroxysmal atrial fibrillation (pAF) show positive results after pulmonary vein isolation [1–3] with freedom from AF recurrence in 70–80% of patients after 1-year [4–6]. Cryoballoon (CB) ablation-mediated PVI is commonly used as primary ablation strategy and was recently demonstrated to be non-inferior compared to the radiofrequency (RF) ablation technique [5, 7]. Vagal activation is an established trigger mechanism of pAF [8, 9] as episodes often occur at night or post exercise—situations that are typically characterized by slow sinus rates [10]. Accordingly, pAF is associated with disorders of the sinoatrial node (SAN) characterized by slow rates and pauses, which appear alternately with atrial tachyarrhythmias, summarized as “bradycardia-tachycardia syndrome” [11]. Furthermore, it is assumed that ~ 50% of patients with sinus node dysfunction are affected by AF, suggesting that both conditions are mutually interrelated [12]. However, little is known how exactly sick sinus syndrome (SSS) affects the atrial myocardium resulting in AF and vice versa.

The finding that autonomic modulation of the intrinsic cardiac nervous system is importantly involved in the generation of AF resulted in the development of ganglionated plexi (GP) ablation, a strategy that primarily aims at the targeted elimination of cardiac neuronal junctions [13–15]. Of note, the most prominent atrial plexi are localized close to the pulmonary veins (PV). However, targeted disruption of GP was shown to result in higher recurrence rates of AF compared to PVI [16]. On the other hand, combination of both PVI and GP ablation was reported to decrease recurrence rates of AF compared to PVI or GP ablation alone [17]. Importantly, GP play a key role for the vagal regulation of the SAN, with the right anterior GP directly mediating neural communication between the SAN and the autonomic nervous system (ANS) [18, 19]. Interestingly, PVI itself results in a rise of SAN rate, suggesting that GP mediating vagal influence on the SAN are compromised by the lesions that are usually produced by PVI [20–23]. In this context, the extent and persistence of sinus rate alteration caused by cryo-PVI and their impact on procedural outcome are still unresolved, while existing data are primarily derived from studies using RF catheter ablation [24, 25]. Here we sought to explore the effects of cryoballoon PVI (cryo-PVI) on sinus rate and its significance for clinical outcomes.

## Methods

### Study population

The study patients were enrolled from a pulmonary vein isolation (PVI) registry at the Heidelberg University Hospital, Germany. The PVI registry and the study protocol were approved by the institutional ethics committee (No. S-585/2016). A total of 110 consecutive patients, who were treated by second-generation 28 mm CB catheter ablation for the first time because of paroxysmal AF, were included. Ninety-one patients could be followed up with respect to freedom from AF after PVI for 1 year, at least. We excluded patients with left ventricular ejection fraction ≤ 40%, left atrial diameter > 50 mm, moderate to severe valvular heart disease or previous heart valve replacement, contraindication for anticoagulation medication, previous cardiac ablation, periprocedural sustained arrhythmia > 30 s, and patients, who received periprocedural application of heart rate-relevant drugs (other than standard protocol using 0.5 mg atropine prior the investigation). With respect to medical history, patients did not change their antiarrhythmic regimen within 3 months prior to the intervention and no patient had discontinued amiodarone within a year prior the procedure. Noteworthy, only patients with stable AAD treatment (including beta-blockers) and patients with no AAD treatment were included in the heart rate analysis. Regardless of whether patients were on AAD therapy or not, included patients received the same therapy throughout the study duration. Patients with change of treatment protocol prior to ablation or during follow-up were excluded from further evaluation. Hence, only patients with stable treatment were analyzed. Prior to the procedure, informed consent was obtained from all patients, in accordance with our study protocol. The study was performed in compliance with the Declaration of Helsinki.

### Study objectives

The study addresses the following objectives: First, we aimed to analyze pre- (1 day prior), peri-, and postinterventional (1 day after) sinus rate behavior and PQ-interval changes of patients treated by CB ablation. Periprocedural rate assessment includes a detailed analysis of rate changes associated with CB treatment of every single PV, as well as immediate pre- and postprocedural sinus rates. Second, we set out to evaluate, whether pre-, peri- or postprocedural sinus rate and/or consecutive rate changes have an effect on procedural outcome with respect to 1-year freedom from AF. Third, we asked, whether rate changes persist over 1 year.

### Cryoballoon ablation procedures

The entire procedure was performed with the patient conscious using a minimal fentanyl-based (0.1–0.3 mg i.v.) analgosedation strategy. Vital parameters such as blood pressure and oxygen saturation were monitored throughout procedure. Every patient received atropine 0.5 mg i.v. prior to procedural onset to prevent vagal reactions, commonly observed in CB ablation. Catheter entries were accessed via the right femoral vein. A steerable diagnostic quadripolar catheter (Xtrem, ELA Medical, Sorin Group, Munich, Germany) was positioned in the coronary sinus. A fluoroscopy-guided, single transseptal approach using a Brockenbrough needle was then followed in all procedures. The surface ECG and bipolar endocardial electrograms were monitored continuously and stored using a digital amplification and recording system (LabSystem™ Pro, Boston Scientific, Malborough, MA, US). After transseptal puncture, a patient weight-adjusted unfractionated heparin bolus was applied i.v. to achieve an activated clotting time (ACT) between 300 and 400 s and titrated infusion of heparin was applied thereafter to reach/maintain this ACT range. For CB ablation a steerable transseptal sheath (12 F, FlexCath™ Advance, Medtronic Inc., Minneapolis, MN, USA) was introduced into the left atrium (LA) over a guidewire to steer the CB (Arctic Front Advance™, 28 mm diameter, Medtronic Inc., Minneapolis, MN, USA). The circular mapping catheter (Medtronic Achieve™, Medtronic Inc., 20 mm diameter, Minneapolis, MN, USA) was inserted through the lumen of the CB. The transseptal sheath was constantly perfused with heparinized 0.9% saline. The CB was guided to all PV ostia. Balloon position and the degree of PV occlusion were evaluated by injection of radiopaque contrast agent diluted in 1:1 ratio with 0.9% saline. As a standard, one delivery of cryo-energy for 3 min, was applied to each PV, according to commonly used protocols recommended for the second generation CB [26]. In case of failure to PV isolation, early recurrence of PV conduction, time to isolation > 40 s, or minimum balloon temperature ≥ 40 °C a 3 min. bonus freeze was administered. An ablation order starting with the left upper PV, continued by the left lower PV, the right upper PV and the right lower PV was applied in all patients included. Before targeting the right-sided PVs, the steerable quadripolar catheter (Xtrem, ELA Medical, Sorin Group) was positioned in the superior vena cava for continuous phrenic nerve stimulation (10 V, 2.9 ms; ~ 50 mA) during cryo-energy application. Delivery of cryo-energy was terminated immediately upon loss of phrenic nerve capture. Conduction block of LA-PV and PV-LA (entrance and exit block) was confirmed by complete elimination of PV potentials or by dissociated electrical PV activity and by using pacing maneuvers aiming for capture within the PV, respectively [27].

### ECG and heart rate assessment

All patients received a pre-procedural 12-lead ECG (after 5 min. at rest) at hospital admission, usually the day before the procedure and were subjected to continuous ECG monitoring for 2 ± 0.5 h prior to the intervention. The intraprocedural monitoring comprised continuous and digitized ECG recording (LabSystem™ Pro, Boston Scientific, Marlborough, MA, US) enabling the evaluation of heart rate parameters and PQ intervals at specified time points throughout the procedure. Generally heart rates were outlined as means of 10 cycle lengths, while episodes with supraventricular or ventricular arrhythmias were discarded. Accordingly, PQ-intervals were depicted as means of 10 consecutive sinus beats. Initial recordings at the onset of the intervention were undertaken 5 min after application of 0.5 mg atropine i.v., but before beginning of instrumentation. For periprocedural acquisition, sinus rates and PQ-intervals were evaluated after CB ablation of every PV. To this end recordings of spontaneous sinus rate activity were analyzed 60 s after deflation of the CB. At the end of the procedure, sinus rate and PQ-parameters were recorded following removal of sheaths and pressure taping. Finally, a postprocedural 12-lead ECG (after 5 min. at rest) as well as continuous ECG monitoring for 6 ± 0.5 h 1 day after the invention were recorded from every patient.

### Clinical follow-up

After hospital discharge patients were encouraged to follow-up visits at our outpatient clinic or their cardiologists’ praxis 1, 3, 6 and 12 months after the procedure. Follow-up visits consisted of a clinical interview, ECGs, and/or 24-h Holter monitoring. Patients were asked to transmit ECGs documentation whenever symptoms of arrhythmia were felt. They were advised to come to our chest-pain unit immediately after arrhythmia onset and to use 24-h Holter monitoring or patient activated event recorder for 2 weeks. A review of arrhythmia symptoms was conducted by telephone interview at 12 months post intervention. Recurrence was defined as any AF or atrial tachyarrhythmia lasting longer than 30 s. Early recurrence of AF (ERAF) was defined as a recurrence within a 3-month blanking period according to the latest guidelines and not considered as recurrence [28, 29]. In case of recurrence, patients were offered a re-do procedure using RF catheter ablation. However, ECG data of patients that had received re-do PVI were excluded from this study. Procedural success was defined as freedom from any recurrence without any post procedural novel onset of antiarrhythmic drug therapy. Of the 110 patients that were evaluated for intraprocedural parameters, 91 could be followed-up for 1 year. 19 patients could not be followed-up because they did not participate in the follow-up visits (6 patients), did not agree with follow-up study participation (1 patient) or changed anti-arrhythmic drugs immediately after intervention (12 patients), precluding from periprocedural heart rate analysis.

### Heart rate follow-up

A systematic mid-term follow-up at 3 months post intervention could be performed in 78 patients using 24-h Holter recording (versus 24-h Holter recording prior to cryo-PVI). A systematic long-term follow-up of sinus rate 1 year after the procedure using 12-lead ECG at rest could be undertaken in 33 participants of the study. Owing to the prerequisite that no change of ant-arrhythmic drugs (AADs) were allowed to qualify for sinus rate follow-up, many patients could not be included.

### Statistical analysis

All primary analyses were blinded to the investigator. Statistical analysis was performed using GraphPad Prism (Version 6.0, Graphpad Software, Inc., San Diego, CA, https://www.graphpad.com). Descriptive statistical analyses included the sample size, arithmetic mean and its standard error for continuous and the sample size, absolute and relative heart rates for categorical variables, separately for each treatment group as well as combined. Visual inspections are performed by means of bar- and line-charts. Comparison between multiple groups was performed using one-way ANOVA followed by a post hoc evaluation of pairwise comparisons using two-tailed Student’s *t* test. Post hoc pairwise comparisons were adjusted using Tukey’s adaption. Fisher’s exact test was used to compare categorical dependent variables with respect to group differences. We investigated into differences in arrhythmia-free survival between the different groups by means of pairwise log-rank tests. All differences were considered significant at a level *p* < 0.05.

## Results

We enrolled 110 patients with paroxysmal AF into the study. Baseline characteristics of patients are depicted in Table 1.

**Table 1:** Baseline characteristics of the study population

Patient characteristics	Value
Patients (*n*)	110
Male gender	75 (68%)
Age (years)	63.73 ± 0.97
Body mass index (kg/m^2^)	27.30 ± 0.42
Left atrial size (mm)	39.85 ± 0.41
LV ejection fraction	
≥ 55%	99 (90%)
45–54%	9 (8%)
30–44%	2 (2%)
AF duration (years)	4.62 ± 0.52
EHRA	2.61 ± 0.05
NYHA	1.32 ± 0.04
Hypertension	79 (72%)
Diabetes mellitus	8 (7%)
Dyslipidemia	51 (46%)
Coronary artery disease	35 (32%)
History of stroke	10 (9%)
CHA_2_DS_2_Vasc-Score	2.07 ± 0.13
Antiarrhythmic medication	
Propafenone	4 (4%)
Flecainide	14 (13%)
Beta blocker	92 (84%)
Amiodarone	7 (6%)
Dronedarone	1 (1%)
Digitalis	6 (6%)

*AF* Atrial fibrillation, *EHRA* European Heart Rhythm Classification, *LV* left ventricular, *NYHA* New York Heart Association Functional Classification

### Intraprocedural effects of cryo-PVI on heart rate

**Fig. 1:**
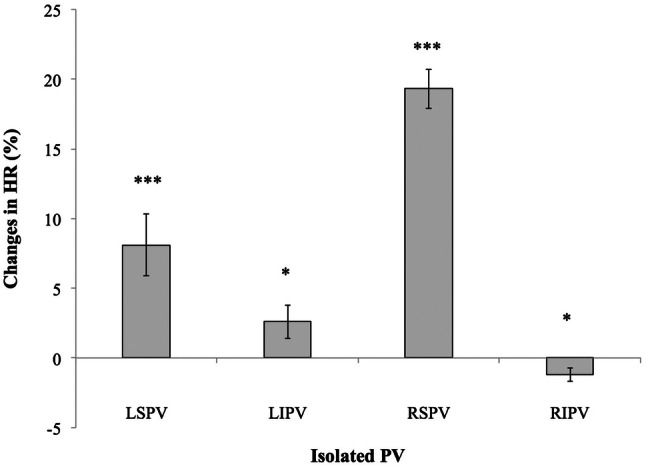
Intraprocedural heart rate changes after isolation of every single pulmonary vein (*n* = 110 patients). *HR* heart rate, *LIPV*: left inferior pulmonary vein, *LSPV*: left superior pulmonary vein, *PV*: pulmonary vein, *RIPV*: right inferior pulmonary vein, *RSPV*: right superior pulmonary vein. * p < 0.05, ** p < 0.01, *** p < 0.001

When comparing heart rate at baseline versus one day after PVI a highly significant rate increase was observed (16.5 ± 1.6%; *p* < 0.001; *n* = 91 patients), which corresponded to an absolute acceleration of rate by 9.8 ± 0.9 beats/min. To further elucidate the mechanisms underlying this effect we analyzed intraprocedural heart rate changes after isolation of every single pulmonary vein (*n* = 110 patients). The most pronounced effect on rate was observed after isolation of the RSPV, which caused a rise of sinus rate by 19.3 ± 1.4% (*p* < 0.001) when compared to the rate prior to RSPV isolation (Fig. 1). Isolation of LSPV and LIPV resulted in relative rises of heart rate by 8.1 ± 2.2% (*p* < 0.001) and 2.6 ± 1.2% (*p* < 0.05), respectively. Isolation of the RIPV, by contrast, was associated with a slight rate decrease (− 1.2 ± 0.5%; *p* < 0.05) (Fig. 1). Procedural parameters are summarized in Table 2.

**Table 2:** Procedural parameters

Parameter	Value
Procedure time (min)	82.31 ± 2.16
Radiation dose (Gy × cm^2^)	9.77 ± 0.57
Ablation duration (min)	16.90 ± 0.64
Ablation duration per vein (min)	4.30 ± 0.17
Ablation duration LSPV (min)	4.54 ± 0.24
Ablation duration LIPV (min)	4.48 ± 0.24
Ablation duration RSPV (min)	4.57 ± 0.24
Ablation duration RIPV (min)	3.51 ± 0.13
Ablation duration per freeze (min)	3.12 ± 0.09

*LIPV* left inferior pulmonary vein, *LSPV* left superior pulmonary vein, *min* minute, *RIPV* right inferior pulmonary vein, *RSPV* right superior pulmonary vein

### Impact of baseline heart rate on cryo-PVI-mediated rate modulation

**Fig. 2:**
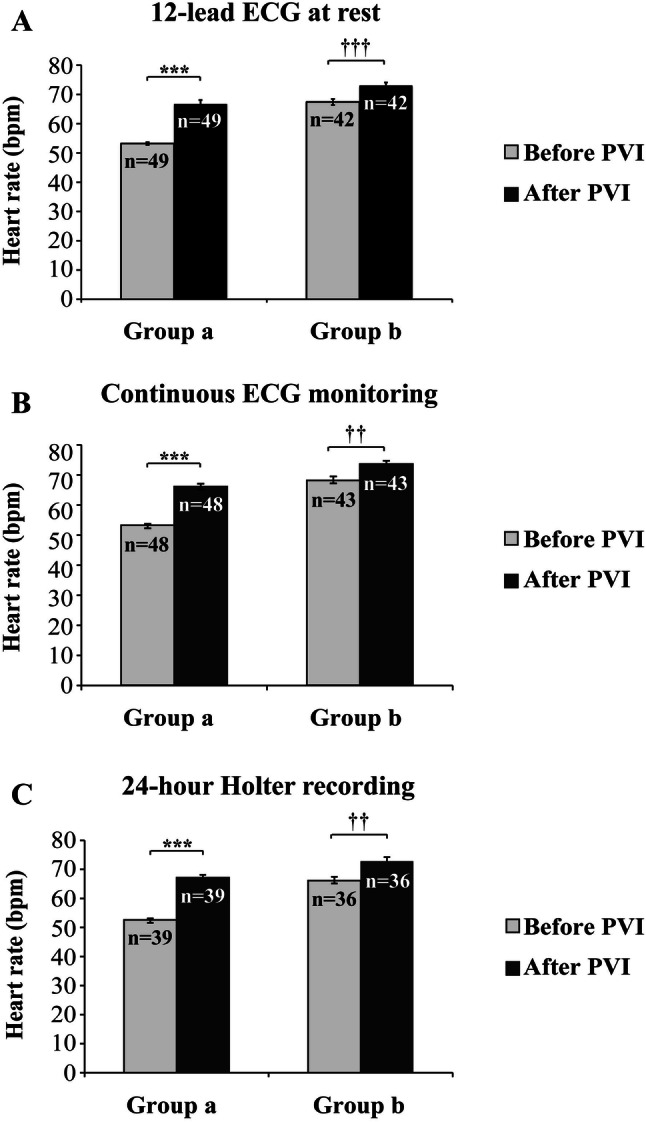
Heart rate modulation in response to cryo-PVI in patients with preinterventional sinus bradycardia (< 60/min., group a, *n* = 49) versus normal sinus rate (≥ 60/min., group b, *n* = 42) documented by **a** ECG at rest, **b** continuous ECG monitoring and **c** 24-h Holter recording. *Bpm* beats per minute, *PVI* pulmonary vein isolation, *n* number of patients. *** p < 0.001, ^†††^* p* < 0.001, ^††^* p* < 0.01

We next asked whether the extent of cryo-PVI-mediated heart rate change is related to pre-interventional baseline heart rate. To address this question the patients of the study were followed-up for 1 year, and divided into a group with pre-interventional sinus bradycardia (group a: < 60 beats/min) and a group with faster heart rate (group b: ≥ 60 beats/min). Patients of group a exhibited a markedly higher rise of sinus rate in response to cryo-PVI (13.5 ± 1.3 beats/min.) than patients of group b (5.6 ± 1.1; *p* < 0.001), when comparing baseline heart rate the day before intervention versus heart rate the day after the intervention (Fig. 2a). To achieve more robust data, results derived from continuous ECG monitoring (pre-interventional versus 1 day post- intervention, total *n* = 91, Fig. 2b) and 24-h Holter recording (pre-intervention versus 3 months post-intervention, total *n* = 78, Fig. 2c) were evaluated according to groups a and b, as well, consistently confirming the effects observed by 12-lead ECG at rest acquisition (Fig. 2a). Interestingly, results from 24-h Holter recording indicate that cryo-PVI mediated effects on rate persist 3 months post-intervention, at least (Fig. 2c, Table 3).

**Table 3:** Follow-up heart rate pattern post cryo-PVI

Time post ablation	Discharge	1 month	3 months	12 months	*p*
Heart rate at rest (beats/min)*	71.7 ± 1.4	70.9 ± 1.3		71.2 ± 1.9	0.93
Minimum heart rate (beats/min)^†^	54.6 ± 0.9		56.8 ± 0.7		0.06
Average heart rate (beats/min)^†^	70.7 ± 1.1		72.4 ± 1.0		0.25
Maximum heart rate (beats/min)^†^	109.7 ± 2.1		114.5 ± 1.7		0.08

Heart rates values are characterized as arithmetic mean ± SEM

*Data set of 33 patients post cryo-PVI evaluated by 12-lead ECG. Statistical analysis by one-way ANOVA

^†^Data set of 75 patients post cryo-PVI evaluated by Holter recording. Statistical analysis by tow-tailed *t* test

### Influence of antiarrhythmic drug (AAD) therapy on cryo-PVI-mediated heart rate modulation

To investigate whether the influence of cryo-induced PVI was related to (stable) therapy with antiarrhythmic drugs, we evaluated heart rate response of patients treated with AADs versus patients without, comparing baseline heart rate the day before versus the day after the intervention. Notably, beta-blocker therapy by far was the most common AAD used (for details please refer to Table 1). Both subgroups showed a significant heart rate increase in response to cryo- PVI (Online Resoure 1). Heart rate values were not significantly different before cryo-PVI (60.9 ± 2.1 versus 59.6 ± 1.0 beats/min. of non-AAD and AAD subgroups, respectively, *p* > 0.05) and not significantly different afterwards (71.7 ± 2.0 versus 69.3 ± 1.0 beats/min. of non-AAD and AAD subgroups, respectively, *p* > 0.05). This indicates that cryo-PVI effect on heart rate may not be influenced significantly by AAD therapy.

### Effects of heart rate on procedural outcome

Among the 91 patients that participated in follow-up investigations within the year after PVI (mean follow-up 12.16 months) 22 patients (24%) were affected by recurrence of AF. To evaluate the influence of heart rate on procedural outcome we investigated the particular effects of pre-procedural heart rate, procedural heart rate change and postprocedural heart rate, respectively. With respect to heart rate acquisition, data were evaluated in triplicate using 12-lead ECG at rest, continuous ECG monitoring and 24-h Holter recording. In the interest of clarity and simplicity results derived from 12-lead ECG data are outlined in the main paper, while the analyses based on the two latter data sets are presented in the Online Resources 2 and 3.

### Impact of baseline heart rate

**Fig. 3:**
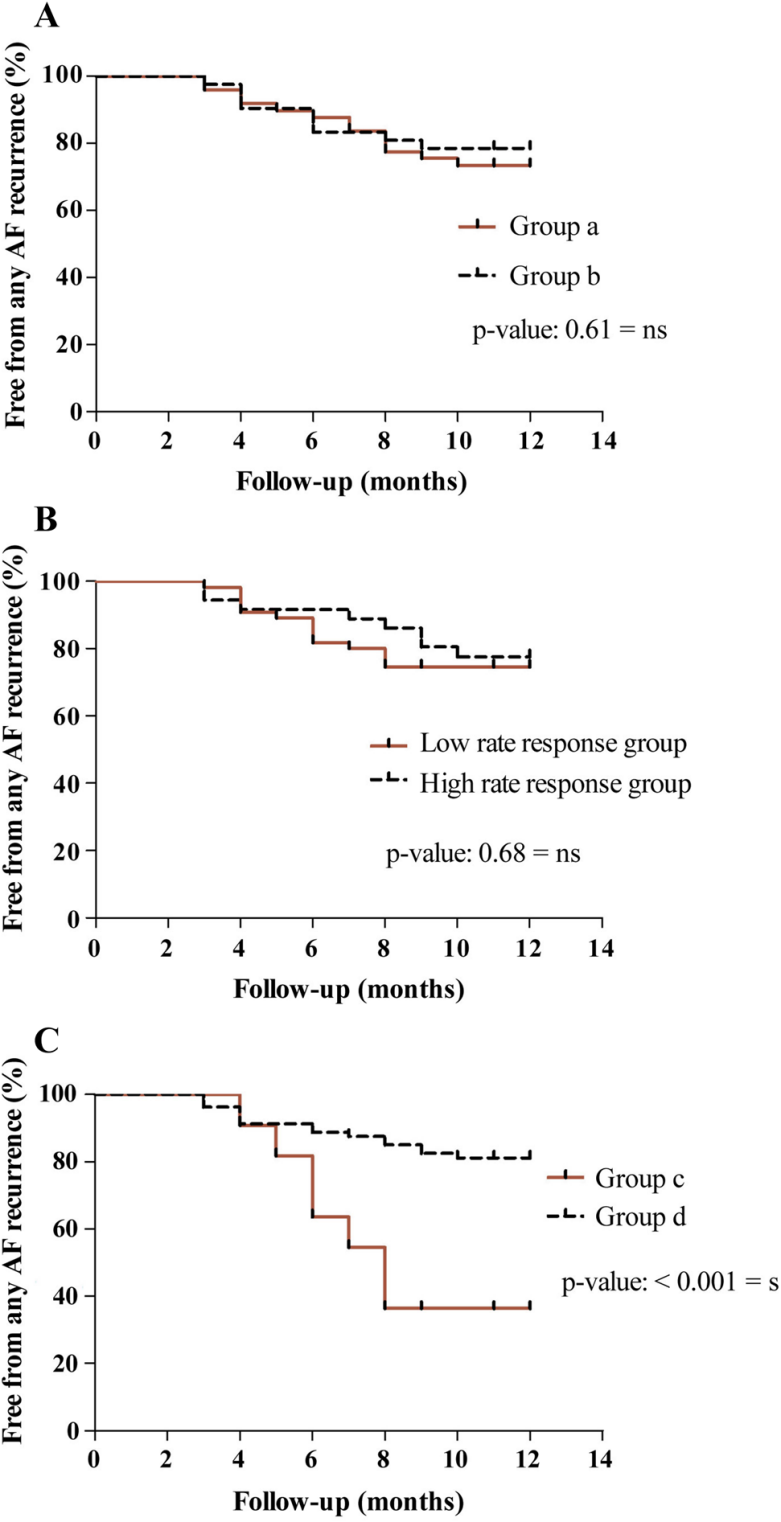
**a** Kaplan–Meier curve of freedom from atrial fibrillation in patients with pre-interventional sinus bradycardia (< 60/min., group a, *n* = 49) and normal sinus rate (≥ 60/min., group b, *n* = 42). **b** Kaplan–Meier curve of freedom from atrial fibrillation in the high rate response. Group (*n* = 36) compared to the low rate response group (*n* = 55). **c** Kaplan–Meier curve of freedom from atrial fibrillation in patients with postinterventional sinus bradycardia (< 60/min., group c, *n* = 11) and normal sinus rate (≥ 60/min., group d, *n* = 80). *AF* atrial fibrillation, *s* significant, *ns* not significant

To analyze the impact of pre-procedural heart rate, study population was divided into groups a and b, as previously described (please refer to 2.). There were no significant differences between patient baseline characteristics of both sub-groups, except for a significantly higher body mass index (BMI) in group b (26.36 ± 0.70 kg/m^2^ versus 28.86 ± 0.76 kg/m^2^; *p* = 0.02) (Online Resource 4). Furthermore, procedural parameters showed no significant differences between the groups (Online Resource 5). Importantly, patients of both groups showed similar results with 13 out of 49 patients of group a versus 9 out of 42 patients of group b being affected by AF recurrence within 1 year after cryo-PVI (*p* > 0.05). Please refer to Kaplan–Meier curves depicted in Fig. 3a. Similar results were observed when employing data derived from continuous ECG monitoring or 24-h Holter recording 3 months post intervention (please refer to Online Resources 2 and 3).

### Impact of procedural rate change

To elucidate whether the extent of procedural rate change predicts procedural outcome study population was divided into two groups. High rate response was defined as > 20% decrease in the cycle length at follow-up 1 day post intervention relative to before the procedure [30, 31]. All other patients were attributed to the low rate response group. With regard to patient baseline characteristics, patients of the high rate response group showed significantly lower baseline heart rate, in line with the results of 2. Furthermore, duration of LIPV cryo-ablation was significantly longer in this group (Online Resources 6 and 7). However, there was no significant difference between both groups with respect to recurrence of AF, with 8 out of 36 patients of the high rate response group affected by recurrence compared to 14 out of 55 patients in the low rate response group (*p* > 0.05) (Fig. 3b). Similar results were observed when employing data derived from continuous ECG monitoring or 24-h Holter recording 3 months post intervention (please refer to Online Resources 2 and 3).

### Impact of postprocedural heart rate

To investigate the influence of postprocedural heart rate on procedural outcome, study population was divided into group c with postinterventional sinus bradycardia (< 60 beats/min) and group d with faster postinterventional heart rate (≥ 60 beats/min). With respect to baseline characteristics, patients of group c were older and had a lower body mass index (BMI) compared to patients of group d (Online Resource 8). Furthermore, mean fluoroscopy time of group c was significantly shorter compared to group d. Of note, duration of cryoablation was not significantly different among groups (Online Resource 9). Remarkably, recurrence rate of patients with post interventional heart rate < 60 beats/min (group c, 0.63) was significantly higher compared to patients with heart rates of ≥ 60/min. (group d, 0.19, *p* < 0.001) (Fig. 3c). Similar results were observed when employing data derived from continuous ECG monitoring or 24-h Holter recording 3 months post intervention (please refer to Online Resources 2 and 3).

### Heart rate analysis throughout the year post cryo-PVI

Prerequisite for participation in mid- and long-term analysis of heart rate after cryo-PVI was the absence of change of any heart rate-modulating drug as well as follow-up visits within the year after the procedure.

**Fig. 4:**
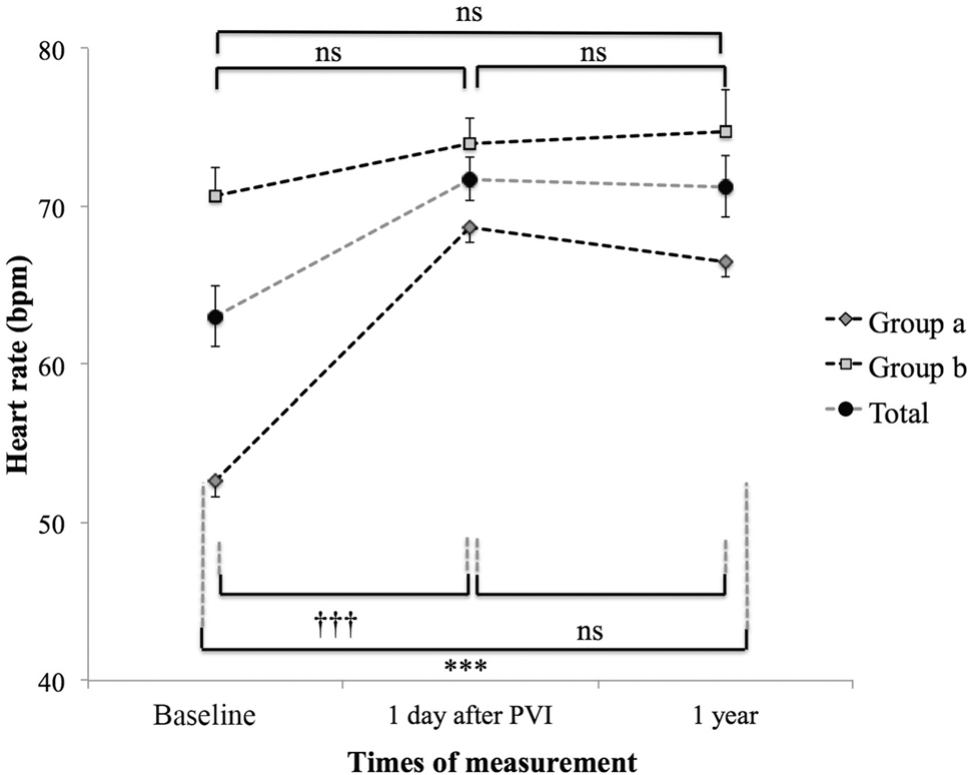
Transition of the heart rate before and after cryo-PVI and 12-months follow-up (*n* = 33 patients). Patients with pre-interventional sinus bradycardia (< 60/min., group a) and normal sinus rates (≥ 60/min., group b) are delineated separately. Total population is denoted by the grey, dashed line in between. HR: heart rate, Ns: not significant, PVI: pulmonary vein isolation. *** p < 0.001, ^†††^* p* < 0.001

75 patients of our original study population were meeting these requirements until the end of the blanking period and participated in Holter recording 3 months post intervention. Mean heart rate at discharge was 70.7 ± 1.1/min. and did not change significantly 3 months later (72.4 ± 1.0/min., *p* > 0.05) (Table 3). However, many patients discontinued the anti-arrhythmic drugs after the blanking period (3 months post intervention), and 33 patients remained with unchanged medical therapy and 12-lead ECG at rest available for evaluation of heart rate pattern 1 and 12 months post intervention. Within this specified population mean heart rate was 63.0 ± 1.9/min. prior to cryo-PVI, rising significantly to 71.7 ± 1.4/min. the day post cryo-PVI (*p* < 0.001). Of note, heart rate remained at this level 1 and 12 months post cryo-PVI (*p* > 0.05) (Table 3, Fig. 4). To study the influence of pre-interventional heart rate on long-term rate behavior post cryo-PVI, groups a and b (previously defined) were compared. Mean pre-interventional heart rate of group a (*n* = 14) was 52.6 ± 0.6/min., rising significantly to 68.7 ± 2.1/min. the day post intervention (*p* < 0.001). One year later mean heart rate remained at the post-interventional level (66.5 ± 2.4/min., *p* > 0.05). Mean heart rate of group b (*n* = 19), by contrast, was 70.7 ± 1.8/min., which increased non-significantly to 74.0 ± 1.7/min. the day post-PVI and was 74.7 ± 2.7/min. 12-months post cryo-PVI (*p* > 0.05 for both) (Fig. 4). An overview of the AAD therapy of patients during follow-up is provided in Online Resource 10 and 11.

### Effects of cryo-PVI on PQ interval

We did not observe any changes of PQ intervals in association with cryo-PVI, neither at intraprocedural measurements after isolation of PVs, nor throughout postprocedural evaluation or 1 year follow-up (for details please refer to Online Resources 12 and 13).

## Discussion

**Fig. 5:**
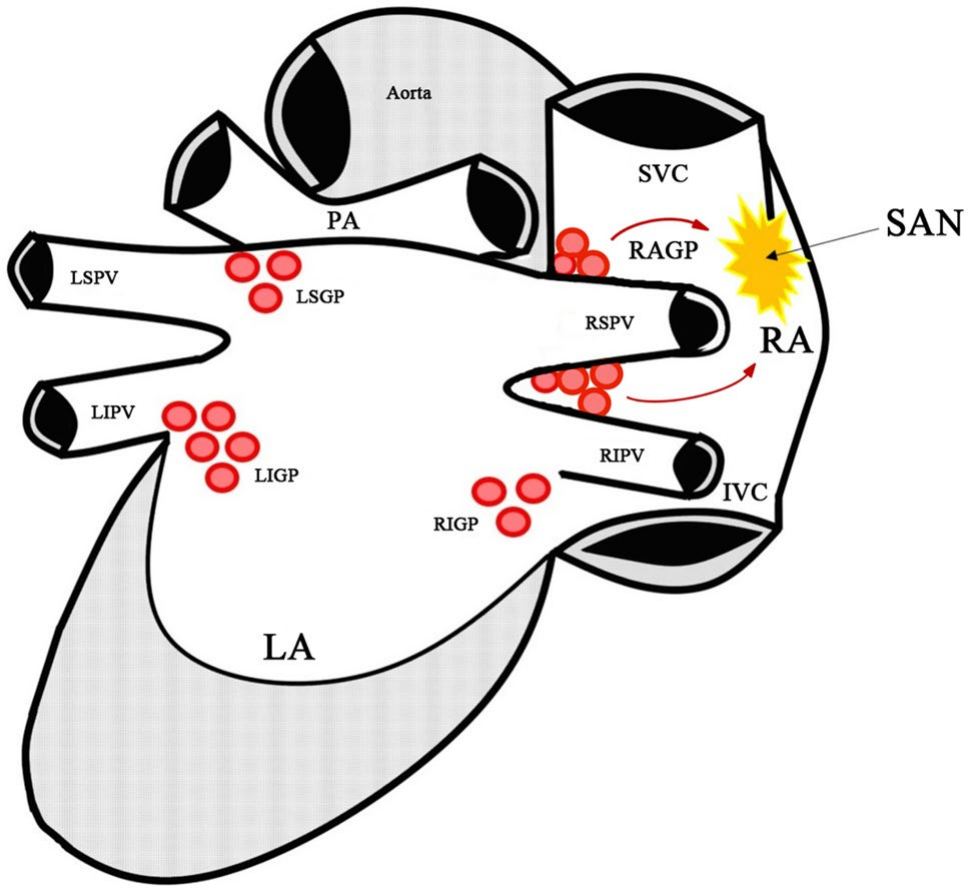
Anatomy of ganglionated plexi related to the pulmonary veins and the sinus node in the left and right atrium. Red arrows depict vagal influence of the right anterior ganglionated plexus on the sinoatrial node. *IVC* inferior vena cava, *LA* left atrium, *LIGP* left inferior ganglionated plexus, *LIPV* left inferior pulmonary vein, *LSGP* left superior ganglionated plexus, *LSPV* left superior pulmonary vein, *PA* pulmonary artery, *RA* right atrium, *RAGP* right anterior ganglionated plexus, *RIGP* right inferior ganglionated plexus, *RIPV* right inferior pulmonary vein, *RSPV* right superior pulmonary vein, *SAN* sinoatrial node, *SVC* superior vena cava

Here we present a real-world single center study investigating the effect of cryo-PVI on sinus rate in patients with paroxysmal AF. The phenomenon of increased heart rate post PVI has been previously described [20–23]. However, given that heart rate importantly influences morbidity and mortality [32], systematic evaluation of this effect after cryo-isolation of every single PV and influence on procedural outcome has not been performed, to our knowledge. We could show that cryo-isolation of the RSPV, by far, caused the most pronounced rate increase. Noteworthy, analysis of rate changes related to isolation of single PVs was evaluated relative to the rate before isolation of a particular PV. Hence, the observed effect may be influenced by the order of PVI [30], which always was LSPV, LIPV, RSPV, RIPV in this study. Nonetheless, impact of PV isolation on sinus rate largely and consistently differed among PVs. The utmost effect of RSPV isolation on sinus rate is in line with previous data [21] and most likely explained by the right anterior GP directly mediating neural communication between the SAN and the ANS [33], co-localizing within the usual lesion of the cryo-balloon at the RSPV ostium (Fig. 5) [18, 19, 34]. Interestingly, total ablation duration and average ablation duration per vein, both showed longer values in patients with higher heart rate increase in our study (Online Resource 7), although statistical significance (*p* < 0.05) was just missed. Thus, longer cryo-ablation duration may favor substrate formation that promotes heart rate increase, e.g. by disruption of GP.

Notably, pre-interventional atropine 0.5 mg i.v. was applied 5 min prior to initial procedural ECG acquisition to effectively suppress adverse vagal reactions, which frequently occur when targeting left superior PV [30]. This standard operating procedure was maintained within our “real world” study, acknowledging that atropine in parts might modulate heart rate response to ablation. However, effects of low dose atropine on heart rate are well known [35], resulting in rate increase within a minute post application, usually followed by slow decline to initial heart rate after 30–60 min. Interestingly, rate often declines further up to 210 min post application, due to paradox central effects of very low atropine doses. Thus, initial atropine application cannot explain increase of heart rate after PV isolation. Moreover, only intraprocedural data were derived under the influence of atropine, while all other results were acquired under atropine-free conditions.

Interestingly, our data reveal that the rise of sinus rate depends on the magnitude of pre-interventional heart rate. In this context, the data also indicate that cryo-PVI effect on heart rate was not influenced significantly by AAD therapy in the patients investigated in this study. Patients with pre-interventional bradycardia exhibited a more pronounced cryo-PVI mediated rate increase compared to patients with initial rates > 60/min. (*p* < 0.001). This may be explained by pre-interventional bradycardia originating from increased intrinsic vagal tone, which is abrogated by cryo-PVI mediated ablation of GP. Thus, the findings suggest a particular benefit of cryo-PVI in patients with bradycardia-tachycardia syndrome, whose medical treatment options often are limited by sinus bradycardia, precluding effective AAD therapy. Such patients, according to our data, experienced significant increase of their basal heart rate by cryo-PVI, and showed similar results in terms of freedom from AF recurrence, compared to patients with normal heart rate.

Given that paroxysmal AF is vagally triggered in many cases [9, 14], we next asked whether pre-interventional heart rate or procedural heart rate change, which indirectly relate to the extent of autonomic influence, are associated with outcome after cryo-PVI. However, none of both parameters showed an association with AF recurrence. By contrast, our data suggest that post-interventional bradycardia, reflected by the group of patients with sinus rates < 60/min. the day post cryo-PVI is linked to an increased risk of AF recurrence in the year post cryo-PVI. Of note, subjects in the post-interventional bradycardia group were older and had lower BMI than those with faster heart rates post cryo-PVI (71.36 versus 63.81 years and 23.18 versus 28.18 kg/m^2^, respectively). In this context, recent studies showed that AF ablation in elderly patients can be performed with success and complication rates similar compared to a younger population [36, 37]. Furthermore, higher body mass index was recently identified as a significant factor associated with atrial tachyarrhythmia recurrence after PVI [38]. Based on these data, it seems unlikely that these two factors significantly contribute to the higher AF recurrence rate in the post-interventional bradycardia group of our study. A possible reason could rather be that in a subset of patients cryo-PVI mediated vagal denervation is less effective resulting in persistence of increased vagal tone and postinterventional bradycardia, providing an explanation for recurrence of vagally-induced AF in this subgroup. Alternatively, primary disease of the sinus node may directly cause a lack of rate response to cryo-PVI in this subgroup. According to this assumption, intrinsic sinoatrial nodal dysfunction (SND), which diminishes responsiveness of the SAN to cryo-PVI mediated disruption of vagal GP, is known to promote electrical and structural remodeling of the conduction system and the atrial myocardium [39], laying the groundwork for recurrence of non-PV dependent AF [40–43].

However, high sinus rate after PVI (≥ 92 beats/min.) was recently associated with a significantly lower clinical recurrence of AF after catheter ablation [24], which corresponds to the observations of our study. According to this line of evidence, postinterventional bradycardia may link to an increased risk of AF recurrence, providing the opportunity to identify patients that require careful follow-up and might need additional treatment.

Previous data from PVI studies using RF catheter ablation provided contradicting results with respect to the persistence of postinterventional rate changes [44–46]. Interestingly, our data, derived from a subpopulation that was followed-up 12 months post cryo-PVI, indicate that effects of cryo-PVI on sinus rate persist throughout the year post intervention, suggesting permanent disruption of vagal influences on the SAN, leading to sustained modification of heart rate. This might be explained by cryo-PVI-mediated lesions that are particularly characterized by larger size and more antral position compared to RF catheter ablation [25].

## Limitations

This is a single center study, with a limited number of patients that were included in a clinical real world scenario. Patient recruitment was confined by the prerequisite that any patient with periprocedural arrhythmia > 30 s, and those, who received periprocedural application of drugs influencing heart rate, were excluded. With respect to the sub-populations available for relating heart rate responses to AF recurrence, a close to complete acquisition of recurrences of AF by permanent rhythm monitoring would increase evidence of the data. Furthermore, continuous AAD administration in parts of the patients, although not changed throughout the follow-up and not significantly different among the investigated groups, might have influenced sinus node behavior. However, although these issues cannot be resolved from the existing data, we believe that the fundamental findings of importance of this study are unlikely to be lost. Moreover, follow-up heart rate data were only applicable for a subpopulation, mainly due to changes of heart rate-relevant medication. Thus, larger cohorts and multi-center studies are needed to validate the data of this study.

## Conclusion

Cryo-PVI causes a significant rise of sinus rate that was primarily driven by isolation of the RSPV. Patients with vagally-induced sinus bradycardia showed the most pronounced heart rate increase with procedural outcome similar to patients with normal heart rates. Thus, the data strengthen cryo-PVI as a primary therapeutic strategy in patients with bradycardia-tachycardia syndrome, which may circumvent or postpone pacemaker implantation and antiarrhythmic drug therapy.

## Electronic supplementary material

Below is the link to the electronic supplementary material.

Supplementary file1 (PDF 995 kb)
