# Supplementary material for: Cryoballoon pulmonary vein isolation-mediated rise of sinus rate in patients with paroxysmal atrial fibrillation
Source: Clin Res Cardiol. 2020 May 13;110(1):124–35. doi: 10.1007/s00392-020-01659-0 (PMC7806555; doi:10.1007/s00392-020-01659-0)
Supplement: Supplementary file 1 — Supplementary file1 (PDF 995 kb) [file 392_2020_1659_MOESM1_ESM.pdf]

Online Material to:

Cryoballoon pulmonary vein isolation-mediated rise of sinus rate in patients with paroxysmal atrial fibrillation

Lara Wagner, BA[a,†]; Fabrice F. Darche, MD[a,†]; Dierk Thomas, MD[a,b]; Patrick Lugenbiel, MD[a]; Panagiotis Xynogalos, MD[a]; Svenja Seide, PhD[c]; Eberhard P. Scholz, MD[a]; Hugo A. Katus, MD[a,b]; and Patrick A. Schweizer, MD[a,b]*

[a]Department of Cardiology, Medical University Hospital Heidelberg, INF 410, D-69120 Heidelberg, Germany.

[b]DZHK (German Centre for Cardiovascular Research), partner site Heidelberg/Mannheim, University of Heidelberg, Germany

[c]Institute of Medical Biometry and Informatics, University of Heidelberg, INF 130.3, D-69120 Heidelberg, Germany.

* Corresponding author

[†] Authors contributed equally to this work

Address for correspondence:

Patrick A. Schweizer, MD

Department of Cardiology, University of Heidelberg, INF 410, D-69120 Heidelberg, Germany

Tel. +49 6221-5639732

Fax +49 6221-565514

E-mail: patrick.schweizer@med.uni-heidelberg.de

Journal: Clinical Research in Cardiology

Online Resource 1: Influence of antiarrhythmic drug (AAD) therapy on cryo-PVI-mediated heart rate modulation

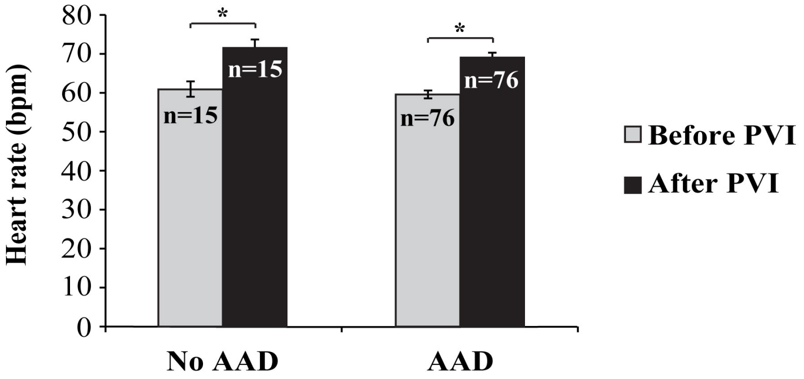

Online Resource 2: Kaplan-Meier curves of freedom from atrial fibrillation. Heart rate analysis by pre- / post-interventional continuous ECG monitoring (n = 91 patients)

A

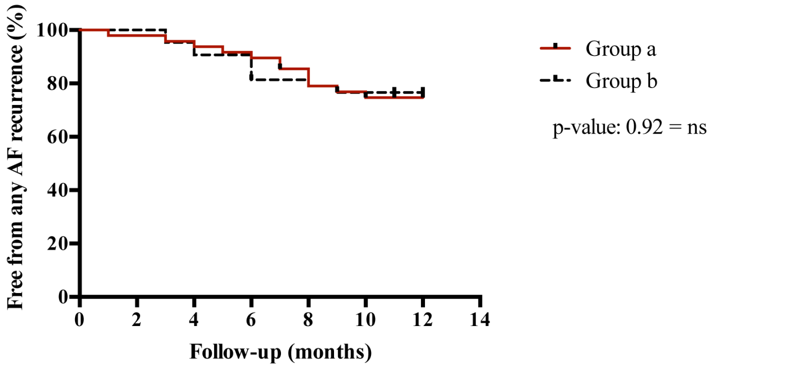

B

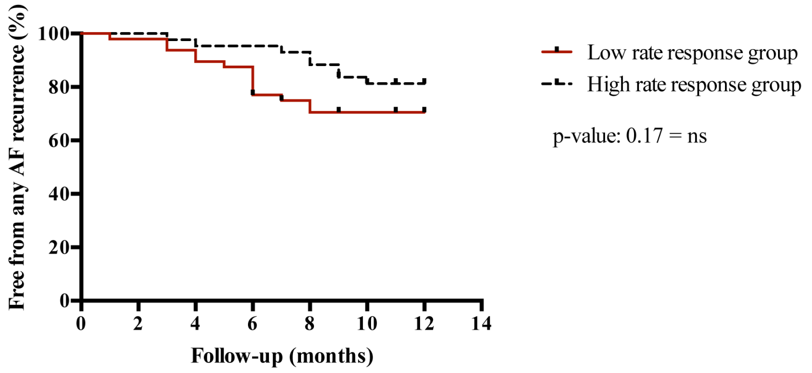

C

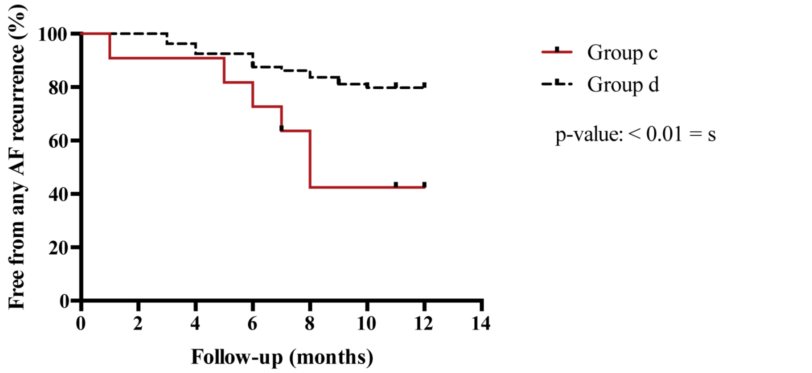

A: Kaplan-Meier curve of freedom from atrial fibrillation in patients with pre-interventional sinus bradycardia (< 60/min., group a) and normal sinus rate (≥ 60/min., group b).
B: Kaplan-Meier curve of freedom from atrial fibrillation in the high rate response group compared to the low rate response group.
C: Kaplan-Meier curve of freedom from atrial fibrillation in patients with postinterventional sinus bradycardia (< 60/min., group c) and normal sinus rate (≥ 60/min., group d).
AF: atrial fibrillation, s: significant, ns: not significant, n: number of analyzed patients.

Online Resource 3: Kaplan-Meier curves of freedom from atrial fibrillation. Heart rate analysis by pre- / post-interventional 24-hour Holter recording (n = 75 patients)

A

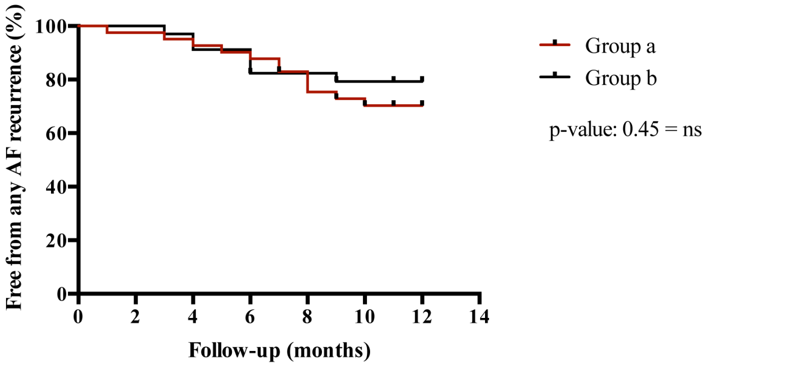

B

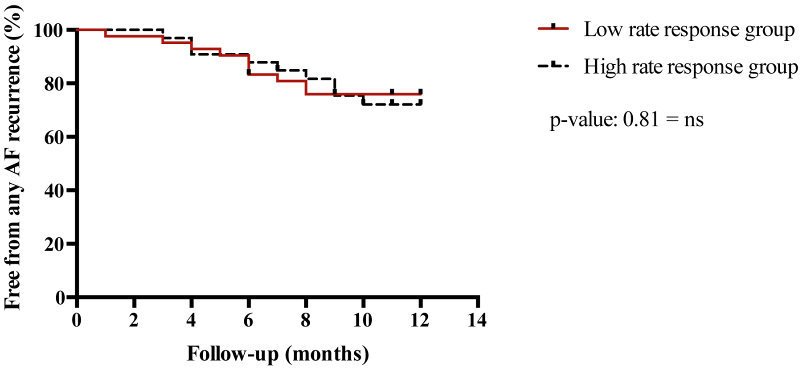

C

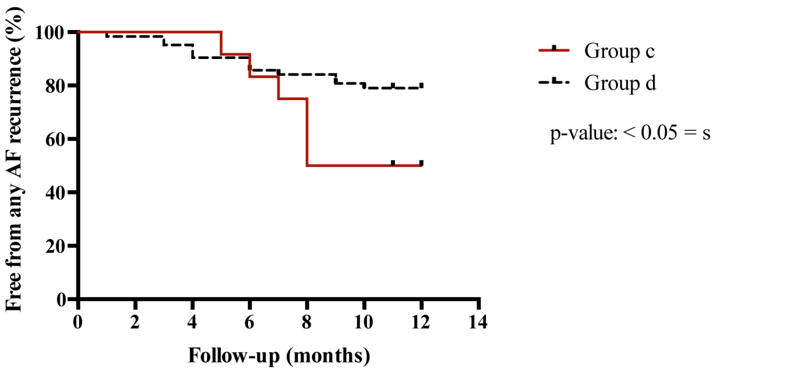

A: Kaplan-Meier curve of freedom from atrial fibrillation in patients with pre-interventional sinus bradycardia (< 60/min., group a) and normal sinus rate (≥ 60/min., group b).
B: Kaplan-Meier curve of freedom from atrial fibrillation in the high rate response group compared to the low rate response group.
C: Kaplan-Meier curve of freedom from atrial fibrillation in patients with postinterventional sinus bradycardia (< 60/min., group c) and normal sinus rate (≥ 60/min., group d).
AF: atrial fibrillation, s: significant, ns: not significant, n: number of analyzed patients.

Online Resource 4: Baseline characteristics of groups a and b

**Patient characteristics**	**Group a**	**Group b**	**p-value**
Patients (n)	49	42	
Male gender	29 (59 %)	30 (71 %)	0.27
Age (years)	65.59 ± 1.28	63.71 ± 1.78	0.38
Body mass index (kg/m^2)	26.36 ± 0.70	28.86 ± 0.76	0.02
Left atrial size (mm)	40.35 ± 0.61	40.21 ± 0.60	0.88
LV ejection fraction			
≥ 55 %	40 (82 %)	37 (88 %)	0.56
45-54 %	8 (16 %)	4 (10 %)	0.37
30-44 %	1 (2 %)	1 (2 %)	1.00
AF duration (years)	4.91 ± 0.68	5.18 (1.00)	0.82
EHRA	2.59 ± 0.08	2.48 ± 0.09	0.33
NYHA	1.26 ± 0.05	1.36 ± 0.07	0.25
Hypertension	36 (74 %)	36 (86 %)	0.20
Diabetes mellitus	5 (10 %)	7 (17 %)	0.56
Dyslipidemia	24 (49 %)	20 (48 %)	1.00
Coronary artery disease	17 (35 %)	14 (33 %)	1.00
History of stroke	3 (6 %)	5 (12 %)	0.46
CHA_2DS_2Vasc-Score	2.29 ± 0.19	2.41 ± 0.23	0.69
Antiarrhythmic medication			
Propafenone	2 (4 %)	0 (0 %)	0.50
Flecainide	4 (8 %)	4 (10 %)	1.00
Beta blocker	41 (84 %)	33 (79 %)	0.60
Amiodarone	2 (4 %)	3 (7 %)	0.66
Digitalis	1 (2 %)	1 (2 %)	1.00

AF: Atrial fibrillation; EHRA: European Heart Rhythm Classification, Group a: patients with pre-interventional sinus bradycardia < 60 beats/min.; Group b patients with faster heart rate (≥ 60 beats/min); LV: left ventricular, NYHA: New York Heart Association Functional Classification

Online Resource 5: Procedural parameters of groups a and b

**Parameter**	**Group a**	**Group b**	**P-value**
HR baseline (min^{-1})	53.22 ± 0.48	67.43 ± 1.04	< 0.001
HR 1 day post PVI (min^{-1})	66.76 ± 1.34	73.07 ± 1.03	< 0.001
Free of recurrence	36 (73 %)	33 (79 %)	0.63
Procedure time (min)	81.82 ± 2.20	88.71 ± 2.89	0.06
Radiation dose (Gy x cm^2)	8.46 ± 0.65	12.57 ± 0.99	< 0.001
Total ablation duration (min)	16.18 ± 0.72	17.5 ± 1.19	0.33
Ablation duration per vein (min)	4.19 ± 0.21	4.35 ± 0.30	0.64
Ablation duration LSPV (min)	4.48 ± 0.36	4.58 ± 0.33	0.83
Ablation duration LIPV (min)	4.48 ± 0.44	4.49 ± 0.59	1.00
Ablation duration RSPV (min)	3.97 ± 0.28	4.88 ± 0.46	0.08
Ablation duration RIPV (min)	3.38 ± 0.20	3.53 ± 0.20	0.59
Time per freeze (min)	3.13 ± 0.18	3.18 ± 0.12	0.84

Group a: patients with pre-interventional sinus bradycardia < 60 beats/min.; Group b patients with faster heart rate (≥ 60 beats/min); HR: heart rate, LIPV: left inferior pulmonary vein, LSPV: left superior pulmonary vein, min: minute, PVI: pulmonary vein isolation, RIPV: right inferior pulmonary vein, RSPV: right superior pulmonary vein

Online Resource 6: Baseline characteristics of high rate response group and low rate response group

**Patient characteristics**	**High rate response group**	**Low rate response group**	**P-value**
Patients (n)	36	55	
Male gender	23 (64 %)	36 (65 %)	1.00
Age (years)	63.36 ± 1.58	65.62 ± 1.43	0.30
Body mass index (kg/m^2)	27.32 ± 0.82	27.65 ± 0.67	0.76
Left atrial size (mm)	39.69 ± 0.79	40.67 ± 0.49	0.27
LV ejection fraction			
≥ 55 %	31 (86 %)	46 (84 %)	1.00
45-54 %	5 (14 %)	7 (13 %)	1.00
30-44 %	0 (0 %)	2 (1 %)	0.52
AF duration (years)	5.27 ± 0.83	4.88 ± 0.81	0.75
EHRA	2.58 ± 0.09	2.51 ± 0.08	0.54
NYHA	1.33 ± 0.07	1.28 ± 0.06	0.57
Hypertension	26 (72 %)	46 (84 %)	0.20
Diabetes mellitus	4 (11 %)	8 (15 %)	0.76
Dyslipidemia	16 (44 %)	28 (51 %)	0.67
Coronary artery disease	11 (31 %)	20 (36 %)	0.65
History of stroke	3 (1 %)	5 (1 %)	1.00
CHA_2DS_2Vasc-Score	2.03 ± 0.21	2.55 ± 0.20	0.08
Antiarrhythmic medication			
Propafenone	2 (5 %)	0 (0 %)	0.15
Flecainide	4 (11 %)	4 (7 %)	0.71
Beta blocker	28 (78 %)	46 (84 %)	0.58
Amiodarone	1 (3 %)	4 (7 %)	0.64
Digitalis	1 (3 %)	1 (2 %)	1.00

AF: Atrial fibrillation, EHRA: European Heart Rhythm Classification, LV: left ventricular, NYHA: New York Heart Association Functional Classification

Online Resource 7: Procedural parameters of high rate response and low rate response group

**Parameter**	**High rate response group**	**Low rate response group**	**P-value**
HR baseline (min^{-1})	53.97 ± 0.74	63.58 ± 1.20	< 0.001
HR 1 day post PVI (min^{-1})	72.33 ± 1.28	67.93 ± 1.23	< 0.05
Free of recurrence	28 (78 %)	41 (75 %)	0.81
Procedure time (min)	82.56 ± 2.88	86.60 ± 2.34	0.28
Radiation dose (Gy x cm^2)	9.78 ± 0.87	10.74 ± 0.84	0.44
Total ablation duration (min)	18.15 ± 1.32	15.90 ± 0.68	0.10
Ablation duration per vein (min)	4.68 ± 0.36	4.00 ± 0.17	0.06
Ablation duration LSPV (min)	4.42 ± 0.46	4.55 ± 0.28	0.81
Ablation duration LIPV (min)	5.48 ± 0.84	3.87 ± 0.23	< 0.05
Ablation duration RSPV (min)	4.76 ± 0.46	4.23 ± 0.32	0.33
Ablation duration RIPV (min)	3.61 ± 0.28	3.31 ± 0.15	0.31
Time per freeze (min)	3.26 ± 0.25	3.09 ± 0.09	0.47

HR: heart rate, LIPV: left inferior pulmonary vein, LSPV: left superior pulmonary vein, min: minute, PVI: pulmonary vein isolation, RIPV: right inferior pulmonary vein, RSPV: right superior pulmonary vein

Online Resource 8: Baseline characteristics of groups c and d

Patient characteristics	Group c	Group d	P-value
Patients (n)	11	80	
Male gender	5 (50 %)	54 (68 %)	0.19
Age (years)	71.36 ± 1.47	63.81 ± 1.16	0.02
Body mass index (kg/m^2)	23.18 ± 0.71	28.18 ± 0.56	0.001
Left atrial size (mm)	40.00 ± 1.05	40.33 ± 0.47	0.81
LV ejection fraction			
≥ 55 %	8 (73 %)	69 (86 %)	0.37
45-54 %	3 (27 %)	9 (11 %)	0.16
30-44 %	0 (0 %)	2 (3 %)	1.00
AF duration (years)	4.20 ± 1.12	5.08 ± 0.65	0.83
EHRA	2.64 ± 0.20	2.53 ± 0.06	0.54
NYHA	1.32 ± 0.12	1.30 ± 0.05	0.89
Hypertension	8 (73 %)	64 (80 %)	0.69
Diabetes mellitus	1 (9 %)	11 (14 %)	1.00
Dyslipidemia	5 (46 %)	39 (49 %)	1.00
Coronary artery disease	4 (36 %)	27 (34 %)	1.00
History of stroke	1 (9 %)	7 (9 %)	1.00
CHA_2DS_2Vasc-Score	2.91 ± 0.37	2.26 ± 0.16	0.15
Antiarrhythmic medication			
Propafenone	0 (0 %)	2 (3 %)	1.00
Flecainide	0 (0 %)	8 (10 %)	0.59
Beta blocker	9 (82 %)	65 (81 %)	1.00
Amiodarone	2 (18 %)	3 (4 %)	0.11
Digitalis	0 (0 %)	2 (3 %)	1.00

EHRA: European Heart Rhythm Classification, Group c: patients with post-interventional sinus bradycardia < 60 beats/min.; Group d patients with faster post-interventional heart rate (≥ 60 beats/min); AF: Atrial fibrillation, LV: left ventricular, NYHA: New York Heart Association Functional Classification

Online Resource 9: Procedural parameters of groups c and d

Parameter	Group c	Group d	P-value
HR baseline (min^{-1})	53.45 ± 2.29	60.65 ± 0.96	0.01
HR 1 day post PVI (min^{-1})	54.27 ± 1.05	71.79 ± 0.78	< 0.001
Free of recurrence	4 (36 %)	65 (81 %)	< 0.01
Procedure time (min)	82.36 ± 5.11	85.36 ± 1.95	0.59
Radiation dose (Gy x cm^2)	5.55 ± 0.51	11.02 ± 0.66	< 0.01
Total ablation duration (min)	15.57 ± 0.73	16.94 ± 0.76	0.54
Ablation duration per vein (min)	3.92 ± 0.18	4.30 ± 0.20	0.69
Ablation duration LSPV (min)	4.79 ± 0.61	4.49 ± 0.27	0.48
Ablation duration LIPV (min)	3.96 ± 0.56	4.56 ± 0.41	0.58
Ablation duration RSPV (min)	4.13 ± 0.93	4.50 ± 0.27	0.67
Ablation duration RIPV (min)	2.93 ± 0.06	3.61 ± 0.17	0.17
Time per freeze (min)	2.93 ± 0.05	3.19 ± 0.13	0.46

Group c: patients with post-interventional sinus bradycardia < 60 beats/min.; Group d patients with faster post-interventional heart rate (≥ 60 beats/min); HR: heart rate, LIPV: left inferior pulmonary vein, LSPV: left superior pulmonary vein, min: minute, PVI: pulmonary vein isolation, RIPV: right inferior pulmonary vein, RSPV: right superior pulmonary vein

Online Resource 10: Antiarrhythmic medication in 75 patients post cryo-PVI subjected to follow-up heart rate analysis evaluated by Holter recording

Antiarrhythmic medication

Propafenone	2 (2.7 %)
Flecainide	7 (9.3 %)
Beta blocker	60 (80 %)
Amiodarone	3 (4 %)
Digitalis	1 (1.3 %)

Online Resource 11: Antiarrhythmic medication in 33 patients post cryo-PVI subjected to follow-up heart rate analysis evaluated by 12-lead ECG at rest

Antiarrhythmic medication

Propafenone	0 (0 %)
Flecainide	1 (0.03 %)
Beta blocker	19 (57.5 %)
Amiodarone	1 (0.03 %)
Digitalis	0 (0 %)

Online Resource 12: Intraprocedural PQ intervals

Time point	Absolute changes (ms)	P-value
post vs. prior cryo-ablation of LSPV	1.31 ± 1.71	> 0.05
post vs. prior cryo-ablation of LIPV	0.80 ± 1.31	> 0.05
post vs. prior cryo-ablation of RSPV	0.80 ± 0.96	> 0.05
post vs. prior cryo-ablation of RIPV	1.19 ± 0.65	> 0.05
Day after PVI vs. day before PVI	1.26 ± 1.35	> 0.05

LIPV: left inferior pulmonary vein, LSPV: left superior pulmonary vein, PVI: pulmonary vein isolation, RIPV: right inferior pulmonary vein, RSPV: right superior pulmonary vein

Online Resource 13: PQ intervals at one year follow-up

Time point	Absolute changes (ms)	P-value
Day after PVI vs. day before PVI	4.38 ± 5.53	> 0.05
1 year post PVI vs. day before PVI	3.05 ± 4.69	> 0.05
1 year post PVI vs. day after PVI	1.33 ± 5.22	> 0.05

PVI: Pulmonary vein isolation
